# Cardiac Troponin I but Not N-Terminal Pro-B-Type Natriuretic Peptide Predicts Outcomes in Cardiogenic Shock

**DOI:** 10.3390/jpm13091348

**Published:** 2023-08-31

**Authors:** Tobias Schupp, Jonas Rusnak, Jan Forner, Kathrin Weidner, Marinela Ruka, Sascha Egner-Walter, Jonas Dudda, Thomas Bertsch, Maximilian Kittel, Michael Behnes, Ibrahim Akin

**Affiliations:** 1 Department of Cardiology, Angiology, Haemastaseology and Medical Intensive Care, University Medical Centre Mannheim, Medical Faculty Mannheim, Heidelberg University, 69117 Heidelberg, Germanymichael.behnes@umm.de (M.B.);; 2 European Center for AngioScience (ECAS) and German Center for Cardiovascular Research (DZHK) Partner Site Heidelberg/Mannheim, 68167 Mannheim, Germany; 3 Institute of Clinical Chemistry, Laboratory Medicine and Transfusion Medicine, Nuremberg General Hospital, Paracelsus Medical University, 90419 Nuremberg, Germany; 4 Institute for Clinical Chemistry, Faculty of Medicine Mannheim, Heidelberg University, 68167 Mannheim, Germany

**Keywords:** cardiogenic shock, cardiac troponin I, NT-proBNP, biomarkers, prognosis, mortality

## Abstract

This study investigates the prognostic value of cardiac troponin I (cTNI) and N-terminal pro-B-type natriuretic peptide (NT-proBNP) levels in patients with cardiogenic shock (CS). Data regarding the prognostic value of cardiac biomarkers in CS is scarce, furthermore, most studies were restricted to CS patients with acute myocardial infarction (AMI). Therefore, consecutive patients with CS from 2019 to 2021 were included. Blood samples were retrieved from day of disease onset (day 1) and on days 2, 3 and 4 thereafter. The prognostic value of cTNI and NT-proBNP levels was tested for 30-day all-cause mortality. Statistical analyses included univariable *t*-tests, Spearman’s correlations, Kaplan–Meier analyses and multivariable Cox proportional regression analyses. A total of 217 CS patients were included with an overall rate of all-cause mortality of 56% at 30 days. CTNI was able to discriminate 30-day non-survivors (area under the curve (AUC) = 0.669; *p* = 0.001), whereas NT-proBNP (AUC = 0.585; *p* = 0.152) was not. The risk of 30-day all-cause mortality was higher in patients with cTNI levels above the median (70% vs. 43%; log rank *p* = 0.001; HR = 2.175; 95% CI 1.510–3.132; *p* = 0.001), which was observed both in patients with (71% vs. 49%; log rank *p* = 0.012) and without AMI-related CS (69% vs. 40%; log rank *p* = 0.005). The prognostic impact of cTNI was confirmed after multivariable adjustment (HR = 1.915; 95% CI 1.298–2.824; *p* = 0.001). In conclusion, cTNI—but not NT-proBNP—levels discriminated 30-day all-cause mortality in CS patients.

## 1. Introduction

Cardiogenic shock (CS) is characterized by ineffective cardiac output resulting in persistent hypotension unresponsive to fluid administration [1,2]. Thereby, CS leads to tissue hypoxia and end-organ failure, which require the need for catecholamine therapy and/or the insertion of mechanical support (MCS) devices [2,3,4,5,6,7]. Despite ongoing improvement of intensive care medicine, CS is still characterized by short-term mortality rates of approximately 50% [8]. Ongoing demographic changes contribute to significant changes in characteristics of patients admitted with CS. Acute myocardial infarction (AMI) still represents the major cause of CS despite improved revascularization strategies and the increasing number of high-volume tertiary centers, however, the number of patients with non-AMI related CS, mainly attributed to acute decompensated heart failure (ADHF), was shown to increase [9,10]. This is of major importance since causal therapies, such as early coronary revascularization, remain limited in this subgroup of patients [8,11,12,13].

Irrespective of the underlying cause leading to CS, the identification of individuals with the highest risk of death remains challenging, and risk prediction models are hard to conduct related to the overall unacceptable high risk of CS-related mortality. Blood-derived biomarkers may represent an easy and feasible method for better risk stratification in CS patients in intensive care units (ICU) [14,15,16,17]. Cardiac biomarkers, such as cardiac troponins and N-terminal pro-B-type natriuretic peptide (NT-proBNP), were shown to predict the risk of all-cause mortality in patients with cardiovascular disease, such as AMI, acute and chronic heart failure (HF) and ventricular tachyarrhythmias, as well as in patients with non-cardiac diseases such as in patients with stroke or sepsis [14,18,19,20,21]. NT-proBNP levels were associated with the risk of all-cause mortality among patients treated in the ICU, however, most of these studies investigated the prognostic value of NT-proBNP combined with cardiac biomarkers that are infrequently measured during routine clinical ICU treatment, such as soluble ST2 or galectin-3 [22,23,24,25]. In line, cardiac troponins (cTN) are released during myocardial injury and are considered for the diagnosis of AMI during routine clinical care [15]. 

Although AMI is the major cause of CS, current research is characterized by limited data regarding the predictive value of cTN and NT-proBNP in CS [17]. In line with this, studies investigating prognosis of CS patients were commonly restricted to patients with AMI or limited by rather small sample sizes, whereas risk prediction models in patients with CS irrespective of underlying trigger mechanisms warrant further investigation [22,26,27]. To the best knowledge of the authors, no study that comprehensively investigated the prognostic value of cTN and NT-proBNP levels is yet available. Therefore, the present study aims to comprehensively investigate the prognostic value of cardiac troponin I (cTNI) compared to NT-proBNP in consecutive patients with CS irrespective of CS etiology.

## 2. Materials and Methods

### 2.1. Study Patients, Design and Data Collection

The present study prospectively included all consecutive patients presenting with CS admitted to the internistic ICU at the University Medical Center Mannheim, Germany, from June 2019 to May 2021, as recently published [28,29]. All relevant clinical data related to the index event were documented using the electronic hospital information system as well as the IntelliSpace Critical Care and anesthesia information system (ICCA, Philips, Philips GmbH Market DACH, Hamburg, Germany) implemented in the ICU, organizing patient data such as admission documents, vital signs, laboratory values, treatment data and consult notes. 

Important laboratory data, ICU-related scores, hemodynamic measurements and ventilation parameters were assessed on the day of admission (i.e., day 1), as well as on days 2, 3 and 4 thereafter. Furthermore, baseline characteristics, prior medical history, length of index hospital stay, data derived from imaging diagnostics, as well as pharmacological therapies were documented. Documentation of source data was performed by intensivists and ICU nurses during routine clinical care. Values of left ventricular ejection fraction (LVEF) and tricuspid annular plane systolic excursion (TAPSE) were retrieved from standardized transthoracic echocardiographic examinations commonly performed during the first 24 h of ICU hospitalization. LVEF measurements were performed in two- and four-chamber apical projections and calculated using the Simpson’s biplane method, and TAPSE was measured in four-chamber apical projections according to the European guidelines [30].

The present study is derived from an analysis of the “Cardiogenic Shock Registry Mannheim” (CARESMA-registry), representing a prospective single-center registry including consecutive patients presenting with cardiogenic shock being acutely admitted to the ICU for internal medicine of the University Medical Center Mannheim (UMM), Germany (clinicaltrials.gov identifier: NCT05575856). The registry was carried out according to the principles of the declaration of Helsinki and was approved by the medical ethics committee II of the Medical Faculty Mannheim, University of Heidelberg, Germany. Because of the registry design with no influence on patient care and solely data assembled in daily clinical routine were used, written informed consent was not required from patients in accordance with the medical ethics committee.

### 2.2. Inclusion and Exclusion Criteria, Study Endpoints

For the present study, all consecutive patients with CS and measurement of cTNI on day 1 were included. No further exclusion criteria were applied. The diagnosis of CS was determined according to the current recommendations of the Acute Cardiovascular Care Association of the European Society of Cardiology [31,32]. Accordingly, cardiogenic shock was defined by hypotension (systolic blood pressure (SBP) < 90 mmHg) for more than 30 min despite adequate filling status or need for vasopressor or inotropic therapy to achieve SBP > 90 mmHg. Additionally, signs for end-organ hypoperfusion must be present such as oliguria with urine output <30 mL/h, altered mental status, cold clammy skin and increased lactate >2 mmol/L. 

Further risk stratification was performed by the presence or absence of AMI as an underlying cause of CS according to current international guidelines [33,34,35]. ST-segment myocardial infarction (STEMI) was defined as a novel rise in the ST segment in at least two contiguous leads with ST-segment elevation ≥2.5 mm in men <40 years, ≥2 mm in men ≥40 years or ≥1.5 mm in women in leads V2–V3 and/or 1 mm in the other leads. Non-ST-segment myocardial infarction (NSTEMI) was defined as the presence of an acute coronary syndrome with a troponin I increase above the 99th percentile of a healthy reference population in the absence of ST-segment elevation but persistent or transient ST-segment depression, inversion or alteration of T wave, or normal ECG, in the presence of a coronary culprit lesion. 

All-cause mortality at 30 days was documented using the electronic hospital information system and by directly contacting state resident registration offices (‘bureau of mortality statistics’). Identification of patients was verified by place of name, surname, day of birth and registered living address. No patient was lost to follow-up with regard to all-cause mortality at 30 days. 

### 2.3. Measurement of cTNI and NT-proBNP

cTNI was measured with the SIEMENS Atellica Solution CH 930™. The lowest detection limit of the assay was 0.015 ng/mL, with a linearity range of 0.025 to 25 ng/mL. The 99th percentile, measured from a healthy reference population, was 0.045 ng/mL, with a coefficient of variation of 10% [20,36]. NT-proBNP determinations were performed as a direct chemiluminescence sandwich immunoassay on the Atellica Solution IM (Siemens Healthineers, Erlangen, Germany). The linear quantification range of the assay for serum and plasma is 35–35,000 pg/mL (4.13–4130 pmol/L). The clinical decision threshold for the NT-proBNP assay to separate healthy from sick patients is 125 pg/mL for patients aged <75 years and 450 pg/mL for patients aged ≥75 years.

### 2.4. Statistical Methods

Quantitative data are presented as mean ± standard error of mean (SEM), median and interquartile range (IQR) and ranges depending on the distribution of the data. They were compared using the Student’s *t* test for normally distributed data or the Mann–Whitney U test for nonparametric data. Deviations from a Gaussian distribution were tested by the Kolmogorov–Smirnov test. Qualitative data are presented as absolute and relative frequencies and were compared using the Chi-square test or the Fisher’s exact test, as appropriate. Box plots for the distribution of cTNI and NT-proBNP levels were created for the comparisons of 30-day survivors and non-survivors on days 1, 2, 3 and on day 4. Spearman’s rank correlation for nonparametric data was used to test for the association of cTNI and NT-proBNP levels with medical and laboratory parameters on day 1.

C-statistics were applied by calculating the receiver operating characteristic (ROC) curves and investigating the corresponding areas under the curves (AUCs) within the entire cohort in order to assess the diagnostic performance of cTNI and NT-proBNP during the course of ICU hospitalization with regard to the 30-day all-cause mortality. The AUCs for the prognostic performance were compared using the method of Hanley et al. [37]. An optimum cut-off value was determined in accordance with the maximum Youden index.

Kaplan–Meier analyses according to the cTNI and NT-proBNP levels on day 1 were performed within the entire study cohort and stratified by patients with AMI- and non-AMI-related CS. Univariable hazard ratios (HRs) were given together with 95% confidence intervals. Multivariable Cox regression models were developed using the “forward selection” option.

Results of all statistical tests were considered significant for *p* ≤ 0.05. SPSS (Version 28, IBM, Armonk, NY, USA) was used for statistics.

## 3. Results

### 3.1. Study Population

From 2019 to 2021, 273 patients with CS were admitted to our institution. A total of 56 patients with no measurement of cTNI on day 1 were excluded. The final study cohort comprised 217 patients with cTNI measurement on day 1. NT-proBNP level on day 1 was measured in 44% (*n* = 96) of these patients. Median cTNI level on day 1 was 0.763 µg/L (IQR 0.164–6.154 µg/L) and median NT-proBNP level was 4089 pg/L (IQR 567–13,523 pg/L), respectively. When stratified by 30-day survivors and non-survivors, patients were median-aged at 72 years and most patients were males (62% vs. 66%; *p* = 0.462) (Table 1). Patients in the non-survivor group were admitted with lower body temperature (35.7 °C vs. 36.0 °C; *p* = 0.034) and higher heart rate (95 bpm vs. 84 bpm; *p* = 0.039). In contrast, the distribution of cardiovascular risk factors, including arterial hypertension (68% vs. 75%; *p* = 0.280), diabetes mellitus (42% vs. 35%; *p* = 0.289), hyperlipidemia (52% vs. 56%; *p* = 0.543) and smoking (39% vs. 36%; *p* = 0.592) were comparable among 30-day non-survivors and survivors. In line with this, the rates of prior coronary artery disease (35% vs. 40%; *p* = 0.696), congestive heart failure (30% vs. 33%; *p* = 0.717), atrial fibrillation (29% vs. 28%; *p* = 0.965) and chronic kidney disease (32% vs. 34%; *p* = 0.789) did not significantly differ among 30-day non-survivors and survivors. Table 2 outlines CS- and ICU-related data, as well as the distribution of laboratory values assessed during routine clinical care. AMI was the most common cause of CS in both groups, whereas AMI was even more common in 30-day non-survivors (59% vs. 43%; *p* = 0.020) compared to survivors. The 30-day non-survivors were admitted with more advanced stages of CS (*p* = 0.001) with higher rates of left ventricular ejection fraction (LVEF) <30% (61% vs. 37%; *p* = 0.001). In line with this, non-survivors required higher doses of norepinephrine on admission (median 0.2 µg/kg/min vs. 0.1 µg/kg/min; *p* = 0.001) and more frequently underwent MCS device insertion during index hospital stay (16% vs. 3%; *p* = 0.003). With regard to laboratory measurements on admission, non-survivors were admitted with higher lactate (4.5 mmol/L vs. 2.7 mmol/L; *p* = 0.001) and creatinine levels (1.58 mg/dL vs. 1.32 mg/dL; *p* = 0.021), higher white blood cell (WBC) counts (15.5 × 10^6^/mL vs. 13.7 × 10^6^/mL; *p* = 0.026) and higher D-dimer levels (16.95 mg/L vs. 6.23 mg/L; *p* = 0.006) (Table 2).

### 3.2. Association of cTNI and NT-proBNP with Clinical and Laboratory Data

Table 3 illustrates the correlations of cTNI and NT-proBNP levels on admission with clinical and laboratory data. On day 1, cTNI levels correlated with WBC count (r = 0.303; *p* = 0.001), whereas no further correlations with biomarkers were observed. However, the cTNI correlated with LVEF (r = 0.305; *p* = 0.001) and with the CS-onset-to-balloon times in patients with AMI-related CS (r = −0.332; *p* = 0.005). In contrast, NT-proBNP levels correlated with age (r = 0.369; *p* = 0.001), creatinine (r = 0.458; *p* = 0.036) and the international normalized ratio (INR) (r = 0.403; *p* = 0.001), as well as C-reactive protein (CRP) (r = 0.555; *p* = 0.001) and procalcitonin (r = 0.355; *p* = 0.010), whereas an inverse correlation with hemoglobin (r = −0.263; *p* = 0.010) was observed. Finally, no correlation of NT-proBNP with cTNI was observed on day 1 (r = −0.028; *p* = 0.787).

### 3.3. Prognostic Performance of cTNI and NT-proBNP Levels

**Figure 1:**
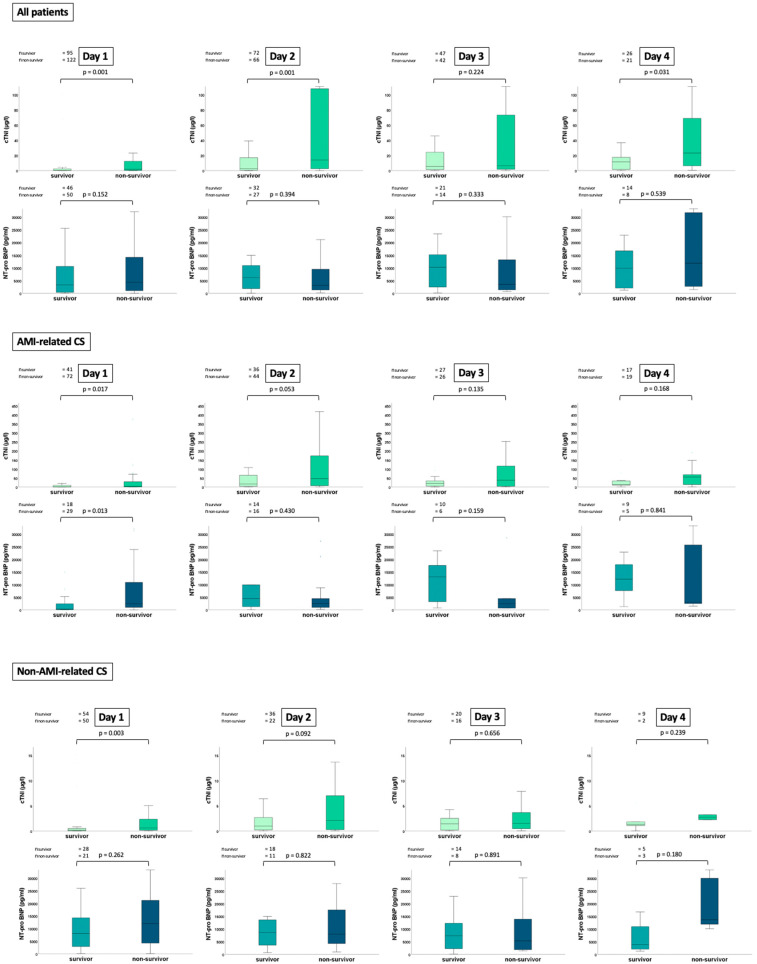
Box plots demonstrating the distribution of cTNI and NT-proBNP levels among patients with CS during the first 4 days of CS onset stratified by 30-day non-survivors and survivors within int entire study cohort (upper panel), as well as stratified by patients with AMI- and non-AMI-related CS (middle and lower panels). Data are presented as the median with interquartile ranges (boxes) and 5–95% percentiles (whiskers).

The median cTNI levels were higher among 30-day non-survivors compared to survivors on day 1 (1.850 µg/L vs. 0.332 µg/L; *p* = 0.001), day 2 (13.916 µg/L vs. 2.733 µg/L; *p* = 0.001) and on day 4 (23.302 µg/L vs. 11.532 µg/L; *p* = 0.031), whereas cTNI levels did not differ on day 3 (6.609 µg/L vs. 5.545 µg/L; *p* = 0.224) (Figure 1; upper panel). On the contrary, NT-proBNP levels did not differ among non-survivors and survivors during the first 4 days of ICU treatment on day 1 (4387 pg/L vs. 3386 pg/L; *p* = 0.152), day 2 (3131 pg/L vs. 6290 pg/L; *p* = 0.394), day 3 (8111 pg/L vs. 10,293 pg/L; *p* = 0.345) and day 4 (11,928 pg/L vs. 9937 pg/L; *p* = 0.570). Of note, cTNI levels on day 1 were higher in non-survivors with AMI- and non-AMI-related CS (Figure 1; middle and lower panels).

**Figure 2:**
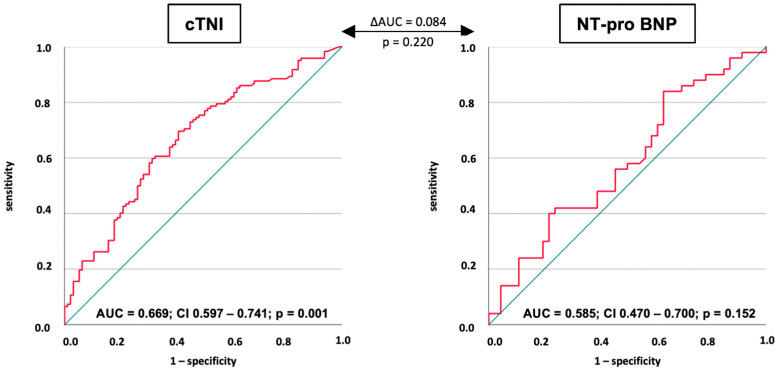
Receiver operating characteristic (ROC) analyses investigating the prognostic performance of cTNI and NT-proBNP for the discrimination of 30-day all-cause mortality within the entire study cohort.

CTNI on day 1 was able to discriminate 30-day mortality (AUC = 0.669; 95% CI 0.597–0.741; *p* = 0.001), whereas NT-proBNP was not (AUC = 0.585; 95% CI 0.470–0.700; *p* = 0.152) (Figure 2). A cTNI level of >0.480 µg/L discriminated the risk of 30-day all-cause mortality with a sensitivity of 69.7% and a specificity of 58.9%. Furthermore, cTNI levels on day 2 (AUC = 0.666; *p* = 0.001) and on day 4 (AUC = 0.685; *p* = 0.031), but not on day 3 (AUC = 0.575; *p* = 0.224), were associated with reliable discrimination of 30-day all-cause mortality (Table 4).

**Figure 3:**
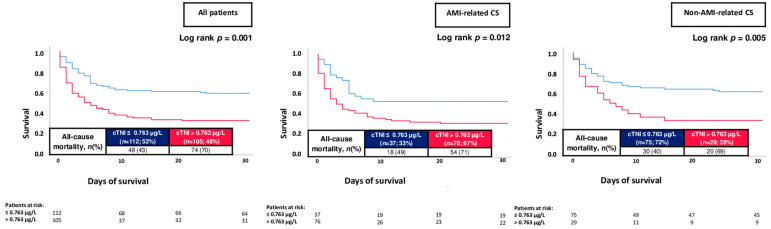
Prognostic impact of cTNI levels on day 1 on the risk of all-cause mortality at 30 days within the entire study cohort (**left panel**), as well as stratified by patients with AMI-related CS (**middle panel**) and non-AMI-related CS (**right panel**).

**Figure 4:**
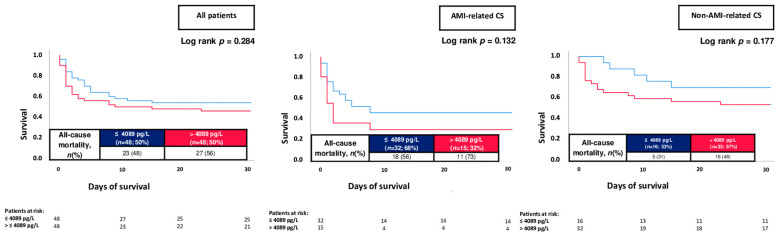
Prognostic impact of NT-proBNP levels on day 1 on the risk of all-cause mortality at 30 days within the entire study cohort (**left panel**), as well as stratified by patients with AMI-related CS (**middle panel**) and non-AMI-related CS (**right panel**).

The overall risk of 30-day all-cause mortality was 56%. When stratified by the median cTNI level on admission, patients with cTNI > 0.763 µg/L had higher risk of 30-day all-cause mortality as compared to patients with lower cTNI levels (70% vs. 43%; log rank *p* = 0.001; HR = 2.175; 95% CI 1.510–3.132; *p* = 0.001) (Figure 3; left panel). Increased mortality rates in patients with elevated cTNI levels were observed both in patients with AMI-related CS (71% vs. 49%; log rank *p* = 0.012; HR = 1.901; 95% CI 1.114–3.246; *p* = 0.014) and non-AMI-related CS (69% vs. 40%; log rank *p* = 0.005; HR = 2.152; 95% CI 1.219–3.799; *p* = 0.008) (Figure 3; middle and right panels). On the contrary, NT-proBNP levels above the median were not associated with the risk of 30-day all-cause mortality within the entire study cohort (56% vs. 48%; log rank *p* = 0.284; HR = 1.336; 95% CI 0.765–2.330; *p* = 0.307), as well as in patients with AMI- (73% vs. 56%; log rank *p* = 0.132; HR = 1.946; 95% CI 0.712–5.323; *p* = 0.195) and non-AMI-related CS (48% vs. 31%; log rank *p* = 0.177; HR = 1.699; 95% CI 0.800–3.610; *p* = 0.168) (Figure 4).

### 3.4. Multivariable Risk Prediction Models

Even after multivariable adjustment, cTNI levels above the median were still associated with the risk of 30-day all-cause mortality (HR = 1.915; 95% CI 1.298–2.824; *p* = 0.001) (Table 5). In addition, increasing age (HR = 1.016; *p* = 0.038), lactate (HR = 1.084; *p* = 0.001), creatinine (HR = 1.138; *p* = 0.030) and the need for cardiopulmonary resuscitation (HR = 1.595; *p* = 0.001) were associated with 30-day all-cause mortality. However, after additional adjustment for NT-proBNP levels, higher NT-proBNP levels were not associated with prognosis in CS (HR = 1.295; *p* = 0.443), whereas cTNI still predicted the risk of 30-day all-cause mortality (HR = 2.822; *p* = 0.002) (Table 5; model 2). Even when entered as a continuous variable, higher cTNI levels were associated with increased risk of 30-day all-cause mortality in CS (HR = 1.002; 95% CI 1.000–1.003; *p* = 0.018).

When stratified by the presence or absence of AMI-related CS, cTNI levels were associated with prognosis both in patients with AMI-related CS (HR = 1.807; 95% CI 1.019–3.202; *p* = 0.043) and non-AMI-related CS (HR = 1.953; 95% CI 1.021–3.735; *p* = 0.043) (Table 6). 

## 4. Discussion

The present study comprehensively investigates the prognostic value of cTNI and NT-proBNP among consecutive CS patients with and without concomitant AMI admitted to an internistic ICU from 2019 to 2021. The main findings of the study can be summarized as follows:

- cTNI levels were consistently higher among 30-day non-survivors as compared to survivors in consecutive CS patients.
- cTNI, but not NT-proBNP levels, were able to discriminate 30-day non-survivors, alongside increased risk of 30-day all-cause mortality in patients with higher cTNI levels.
- The negative prognostic impact of increased cTNI levels was demonstrated irrespective of AMI- or non-AMI-related CS and confirmed even after multivariable adjustment.

Natriuretic peptides, such as BNP and NT-proBNP, represent quantitative biomarkers to reflect the presence and severity of cardiac stress and HF, which were shown to be increased in patients with high wall stress, cardiac filling pressure and intra-cardiac volume. Related to their high diagnostic accuracy for HF, the measurement of natriuretic peptides is recommended in patients with HF-related symptoms [38]. In addition to HF, NT-proBNP levels may be increased in patients with acute cardiovascular diseases, including AMI, pulmonary embolism or atrial fibrillation [39,40,41]. In contrast, limited data regarding the prognostic significance of NT-proBNP levels in CS are yet available, with conflicting findings, whereas most studies were limited by a small sample size. 

Katayama et al. identified BNP as an independent predictor of all-cause mortality among 42 patients with AMI-related CS [42]. In line with this study, NT-proBNP was shown to indicate poor prognosis in 58 CS patients, especially when combined with interleukin-6. Thus, NT-proBNP levels were specifically shown to correlate with prognosis in patients with successful coronary revascularization [43]. The prognostic impact of NT-proBNP was confirmed by Sharma et al. in 42 patients with STEMI-related CS, suggesting reliable prediction of in-hospital mortality (AUC = 0.748) [26], which may be attributed to more advanced stages of left ventricular dysfunction but may be aggravated by prevalent acute kidney injury or concomitant sepsis. Thus, NT-proBNP may be increased as a consequence of concomitant septic cardiomyopathy, type 2 AMI or drug toxicity, however, our study group recently suggested NT-proBNP levels did not differ among sepsis survivors and non-survivors, alongside with a poor predictive accuracy with regard to 30-day all-cause mortality in 162 patients with sepsis and septic shock [20,44]. Within the present study, NT-proBNP levels correlated with inflammatory markers such as CRP and WBC count, and more than half of the patients suffered from concomitant infectious disease. However, NT-proBNP was a poor predictor of 30-day all-cause mortality within the present study including consecutive CS patients with and without AMI. This is in line with a study by Pöss et al. including 51 CS patients demonstrating NT-proBNP levels failed to predict outcomes following CS [45]. However, the generalizability of these studies may be limited related to the rather small sample size. Even in the present study including consecutive CS patients on admission, NT-proBNP levels were infrequently measured in only 44% of the included patients with cTNI measurement, which may have influence on the lack of the prognostic impact of NT-proBNP measurement. Of note, the present study is still one of first studies to investigate the predictive value of NT-proBNP in consecutive CS patients. Furthermore, the present study—for the first time—demonstrated the prognostic value of cTNI was superior compared to the predictive value of NT-proBNP. 

Recently, the prognostic value of a risk score based on blood-derived biomarkers was developed within a sub-study of the CULPRIT-SHOCK trial. Ceglarek et al. created the so-called “CLIP” score, including cystatin C, lactate, interleukin-6 and NT-proBNP, to predict the risk of all-cause mortality following AMI-related CS, including 458 from a total of 706 patients originally enrolled in the CULPRIT-SHOCK trial. However, important pre-selection may be present, including only patients with AMI-related CS with concomitant multi-vessel disease. Furthermore, specifically cystatin C and interleukin-6 are not routinely measured in CS patients, whereas no distinct sub-analyses were performed regarding the prognostic role of cardiac biomarkers in CS [46]. The major strength of the present study lies in the consecutive recruitment of CS patients irrespective of underlying CS etiology. Although AMI was reported to be the main cause of CS within our study, 48% suffered from CS not related to AMI. CTN is characterized by a high sensitivity and specificity for the detection of myocardial necrosis and is embedded in the definition and decision-making of AMI [31,33,47,48]. Data from the “Global Registry of Acute Coronary Events” demonstrated increased risk of cardiac arrest, ventricular tachyarrhythmias, CS and moreover mortality in patients with increased cTN levels in more than 16,000 with non-ST-segment elevation acute coronary syndrome [49]. Even in patients with STEMI, elevated cTN levels were shown to indicate worse clinical outcomes including higher risk of all-cause mortality [50]. Furthermore, higher cTNI levels were associated with severity and progression of CAD [51].

Although it was demonstrated that increased cTN levels may increase the risk CS, very limited data are available focusing on the prognostic value of cTN levels in patients admitted with CS even though CS occurs in up to 10% of patients with AMI [52,53]. The association of cTNT levels in CS patients with concomitant VA-ECMO treatment was investigated by Li et al. within 72 patients. CTNT levels were significantly higher among non-survivors compared to survivors on days 2 and 3, whereas specifically the cTNT decline rate was associated with reliable prediction of ICU mortality [27]. Of note, Lim et al. investigated the prognostic role of myocardial ischemia, taking into account cTN levels, ECG and echocardiographic data, in 93 patients admitted to an ICU. They demonstrated that myocardial ischemia, diagnosed by a multi-modal approach, was present in one out of four ICU patients and associated with increased ICU mortality. In contrast, elevated cTN levels alone were not associated with outcomes, however, their study was performed including a general ICU population [54]. The present study is—to the best knowledge of the authors—the first that investigated the prognostic role of cTNI levels in consecutive CS patients with a reliable sample size. Interestingly, cTNI was able to discriminate 30-day mortality in CS patients, both in patients with AMI- and non-AMI-related CS. Therefore, the measurement of cTNI may be helpful to improve prediction of short-term outcomes following CS, which are still characterized by an unacceptable high risk of death.

This study has several limitations. Due to the single-center and observational study design, results may be influenced by measured and unmeasured confounding variables. For the present study, no sequential cTNI measurement during day 1 of CS, respectively, AMI was assessed. Furthermore, NT-proBNP was only measured in 44% of patients with cTNI measurement, which may limit the generalizability of the study. Related to the hemodynamic instability in the setting of CS, pre-existent treatment with heart failure pharmacotherapies was discontinued in the initial phase of CS, whereas the exact duration of discontinuation was not assessed for the present study. Finally, no information on long-term mortality was available for the present study.

## 5. Conclusions

In conclusion, the present study demonstrates for the first time that cTNI—but not NT-proBNP—was able to discriminate 30-day mortality in CS patients. Interestingly, elevated cTNI levels were associated with impaired risk of 30-day mortality in the presence or absence of AMI-related CS. The negative prognostic impact of cTNI was confirmed even after multivariable adjustment.

## Figures and Tables

**Table 1:** Baseline characteristics.

	Survivor (*n* = 95)		Non-Survivor (*n* = 122)		*p* Value
**Age, median; (IQR)**	**71**	(61–79)	73	(63–81)	0.409
**Male sex**, *n* (%)	63	(66.3)	75	(61.5)	0.462
**Body mass index**, kg/m^2^ (median, (IQR))	26.2	(24.2–29.4)	26.8	(24.5–30.5)	0.380
**Vital signs on admission**, (median, (IQR))					
Body temperature (°C)	36.0	(35.3–36.6)	35.7	(34.4–36.5)	**0.034**
Heart rate (bpm)	84	(69–104)	95	(72–113)	**0.039**
Systolic blood pressure (mmHg)	110	(94–131)	111	(93–131)	0.432
Respiratory rate (breaths/min)	19	(16–22)	20	(17–24)	0.161
**Cardiovascular risk factors**, *n* (%)					
Arterial hypertension	71	(74.7)	83	(68.0)	0.280
Diabetes mellitus	33	(34.7)	51	(41.8)	0.289
Hyperlipidemia	53	(55.8)	63	(51.6)	0.543
Smoking	34	(35.8)	48	(39.3)	0.592
**Prior medical history**, *n* (%)					
Coronary artery disease:	38	(40.0)	43	(35.2)	0.696
Congestive heart failure	31	(32.6)	37	(30.3)	0.717
Atrial fibrillation	27	(28.4)	35	(28.7)	0.965
Chronic kidney disease	32	(33.7)	39	(32.0)	0.789
Stroke	11	(11.6)	11	(9.0)	0.535
COPD	16	(16.8)	29	(23.8)	0.212
Liver cirrhosis	4	(4.2)	3	(2.5)	0.469
**Prior medical history**, *n* (%)					
ACE-inhibitor	34	(35.8)	37	(30.3)	0.395
ARB	18	(18.9)	19	(15.6)	0.512
Beta-blocker	48	(50.5)	54	(44.3)	0.359
ARNI	4	(4.2)	1	(0.8)	0.099
Aldosterone antagonist	14	(14.7)	16	(13.1)	0.731
Diuretics	35	(36.8)	50	(41.0)	0.535
ASA	29	(30.5)	34	(27.9)	0.669
P2Y12-inhibitor	9	(9.5)	11	(9.0)	0.908
Statin	47	(49.5)	46	(37.7)	0.082

ACE, angiotensin-converting enzyme; ARB, angiotensin receptor blocker; ARNI, angiotensin receptor neprilysin inhibitor; ASA, acetylsalicylic acid; COPD, chronic obstructive pulmonary disease; IQR, interquartile range. Level of significance *p* < 0.05; bold type indicates statistical significance.

**Table 2:** Shock-related and follow-up data.

	Survivor (*n* = 95)		Non-Survivor (*n* = 122)		*p* Value
**Cause of CS, *n* (%)**					
Acute myocardial infarction (AMI)	41	(43.2)	72	(59.0)	**0.020**
Arrhythmic	20	(21.1)	7	(5.7)	**0.001**
ADHF	20	(21.1)	29	(23.8)	0.635
Pulmonary embolism	4	(4.2)	9	(7.4)	0.329
Valvular	3	(3.2)	2	(1.6)	0.656
Cardiomyopathy	4	(4.2)	3	(2.5)	0.702
Pericardial tamponade	3	(3.2)	0	(0.0)	0.082
**Onset of cardiogenic shock**					
Primary CS, *n (%)*	70	(73.7)	83	(68.0)	0.365
AMI-onset-balloon-time, min (median, (IQR))	164	(123–256)	157	(119–208)	0.474
CS-onset-balloon-time, min (median, (IQR))	146	(93–193)	157	(118–201)	0.308
Secondary CS, *n (%)*	25	(26.3)	39	(32.0)	0.365
**Coronary angiography**, *n* (%)	75	(79.8)	87	(71.3)	0.154
No evidence of CAD	12	(16.0)	8	(9.2)	0.189
1-vessel disease	16	(21.3)	14	(16.1)	0.392
2-vessel disease	13	(17.3)	15	(17.2)	0.988
3-vessel disease	34	(45.3)	50	(57.5)	0.123
Left main trunk	9	(12.0)	8	(9.2)	0.561
Left anterior descending	36	(48.0)	57	(65.5)	**0.025**
Right coronary artery	36	(48.0)	45	(51.7)	0.636
Left circumflex	26	(34.7)	50	(57.5)	**0.004**
**PCI**, *n* (%)	41	(43.2)	67	(54.9)	0.086
Number of stents, (median; (IQR))	75	(79.8)	87	(71.3)	0.154
CABG, *n* (%)	12	(16.0)	8	(9.2)	0.189
Chronic total occlusion, *n* (%)	16	(21.3)	14	(16.1)	0.392
**Infection**, *n* (%)	13	(17.3)	15	(17.2)	0.988
**Classification of CS**, *n* (%)					
Stage A	0	(0.0)	0	(0.0)	**0.001**
Stage B	5	(5.3)	0	(0.0)	
Stage C	40	(42.1)	31	(25.4)	
Stage D	8	(8.4)	8	(6.6)	
Stage E	42	(44.2)	83	(68.0)	
**Transthoracic echocardiography**					
LVEF >55%, (*n*, %)	12	(13.2)	11	(9.7)	0.438
LVEF 54–41%, (*n*, %)	17	(18.7)	9	(8.0)	**0.023**
LVEF 40–30%, (*n*, %)	28	(30.8)	24	(21.2)	0.121
LVEF <30%, (*n*, %)	34	(37.4)	69	(61.1)	**0.001**
LVEF not documented, (*n*, %)	4	-	9	-	-
VCI, cm (median, (IQR))	1.8	(1.4–2.2)	1.9	(1.6–2.2)	0.334
TAPSE, mm (median, (IQR))	17	(12–20)	14	(11–17)	0.133
**Cardiopulmonary resuscitation**					
OHCA, *n* (%)	35	(36.8)	60	(49.2)	0.069
IHCA, *n* (%)	7	(7.4)	23	(18.9)	**0.015**
Shockable rhythm, *n* (%)	32	(33.7)	37	(30.3)	0.598
Non-shockable rhythm, *n* (%)	63	(66.3)	85	(69.7)	0.598
ROSC, min (median, IQR)	11	(5–19)	19	(12–31)	**0.001**
**Respiratory status**					
Mechanical ventilation, *n* (%)	50	(52.6)	85	(69.7)	**0.010**
Duration of mechanical ventilation, days, (mean, (IQR))	2	(0–8)	2	(1–6)	0.196
PaO_2_/FiO_2_ ratio, (median, (IQR))	230	(147–367)	214	(122–331)	0.403
PaO_2_, mmHg (median, (IQR))	103	(80–162)	103	(77–168)	0.919
**Multiple-organ support during ICU**					
Norepinephrine on admission, µg/kg/min (median, (IQR))	0.1	(0.0–0.1)	0.2	(0.1–0.6)	**0.001**
Dobutamine, *n (%)*	19	(20.0)	48	(39.3)	**0.002**
Levosimendan, *n (%)*	16	(16.8)	42	(34.4)	**0.004**
Mechanical circulatory assist device, *n* (%)	3	(3.2)	19	(15.6)	**0.003**
**Baseline laboratory values**, (median, (IQR))					
pH	7.30	(7.23–7.36)	7.27	(7.17–7.36)	0.056
Lactate (mmol/L)	2.7	(1.6–4.2)	4.5	(2.2–9.7)	**0.001**
Sodium (mmol/L)	139	(136–141)	138	(136–141)	0.600
Potassium (mmol/L)	4.2	(3.7–4.9)	4.4	(3.8–5.0)	0.574
Creatinine (mg/dL)	1.32	(1.10–1.75)	1.58	(1.21–2.27)	**0.021**
Hemoglobin (g/dL)	12.7	(10.6–14.6)	12.5	(10.8–13.9)	0.428
WBC (10^6^/mL)	13.67	(10.00–17.72)	15.49	(12.16–19.86)	**0.026**
Platelets (10^6^/mL)	225	(171–287)	224	(175–265)	0.587
INR	1.13	(1.04–1.29)	1.19	(1.10–1.41)	**0.001**
D-dimer (mg/L)	6.23	(2.36–15.08)	16.95	(3.64–32.00)	**0.006**
AST (U/L)	116	(37–228)	171	(64–488)	**0.038**
ALT (U/L)	60	(30–149)	96	(39–312)	**0.048**
Bilirubin (mg/dL)	0.58	(0.40–1.02)	0.62	(0.43–0.94)	0.456
Troponin I (µg/L)	0.332	(0.087–2.494)	1.850	(0.344–12.431)	**0.001**
NT-proBNP (pg/mL)	3386	(407–10,824)	4387	(1090–15,627)	0.152
Procalcitonin (ng/mL)	0.25	(0.06–0.60)	0.28	(0.17–1.52)	0.314
CRP (mg/L)	6	(4–28)	13	(4–35)	0.335
**Follow up data**, *n* (%)					
ICU time, days (median, (IQR))	4	(3–11)	3	(2–6)	**0.001**

ADHF, acute decompensated heart failure; ALT, alanine aminotransferase; AMI, acute myocardial infarction; AST, aspartate aminotransferase; CABG, coronary artery bypass grafting; CRP, C-reactive protein; CS, cardiogenic shock; GFR, glomerular filtration rate; ICU, intensive care unit; IHCA, in-hospital cardiac arrest; INR, international normalized ratio; IQR, interquartile range;; NT-proBNP, aminoterminal pro-B-type natriuretic peptide; OHCA, out-of-hospital cardiac arrest; PCI, percutaneous coronary intervention; ROSC, return of spontaneous circulation; STEMI, ST-elevation myocardial infarction; TAPSE, tricuspid annular plane systolic excursion; VCI, vena cava inferior; WBC, white blood cells. Level of significance *p* < 0.05; bold type indicates statistical significance.

**Table 3:** Correlations of cTNI and NT-proBNP with laboratory and clinical parameters in all patients on day 1.

	cTNI		NT-proBNP	
	r	*p* Value	r	*p* Value
Age	−0.017	0.805	0.369	** 0.001 **
Body mass index (kg/m^2^)	−0.006	0.935	0.054	0.602
Heart rate (bpm)	0.115	0.095	−0.077	0.454
Systolic blood pressure (mmHg)	0.064	0.356	−0.200	0.051
Norepinephrine (µg/kg/min)	0.054	0.437	0.045	0.666
Lactate (mmo/L)	0.101	0.140	0.093	0.369
Creatinine (mg/dL)	−0.050	0.467	0.458	** 0.001 **
Hemoglobin (g/dL)	0.113	0.097	−0.263	** 0.010 **
WBC count (10^6^/mL)	0.303	** 0.001 **	−0.136	0.187
Platelet count (10^6^/mL)	0.041	0.548	−0.124	0.228
INR	0.074	0.287	0.403	** 0.001 **
cTNI (µg/L)	-	-	−0.028	0.787
NT-proBNP (pg/mL)	−0.028	0.787	-	-
Procalcitonin (ng/mL)	0.122	0.337	0.355	** 0.010 **
CRP (mg/L)	0.001	0.986	0.555	** 0.001 **
LVEF (%)	0.305	** 0.001 **	0.281	** 0.006 **
TAPSE (mm)	−0. 018	0. 889	−0. 425	** 0. ** ** 007 **
AMI-onset-balloon-time (min)	−0. 173	0. 151	0. 408	0. 053
CS-onset-balloon-time (min)	−0.332	** 0.005 **	0.468	** 0.024 **

AMI, acute myocardial infarction; CRP, C-reactive protein; CS, cardiogenic shock; cTNI, cardiac troponin I; INR, international normalized ratio; LVEF, left ventricular ejection fraction; NT-proBNP, aminoterminal pro-B-type natriuretic peptide; TAPSE, tricuspid annular plane systolic excursion; WBC, white blood cells. Level of significance *p* < 0.05; bold type indicates statistical significance.

**Table 4:** C-tatistics for the discrimination of 30-day all-cause mortality. Data are presented as area under the curve (95% CI).

	cTNI	NT-pro BNP	*p* Value
**Day 1**	0.669 (0.597–0.741) *p* = **0.001**	0.585 (0.470–0.700) *p* = 0.152	0.220
**Day 2**	0.666 (0.576–0.756) *p* = **0.001**	0.435 (0.286–0.584) *p* = 0.394	**0.009**
**Day 3**	0.575 (0.454–0.695) *p* = 0.224	0.405 (0.210–0.599) *p* = 0.333	0.131
**Day 4**	0.685 (0.527–0.843) *p* = **0.031**	0.580 (0.320–0.840) *p* = 0.539	0.492

cTNI, cardiac troponin I; NT-proBNP, aminoterminal pro-B-type natriuretic peptide. Level of significance *p* < 0.05; bold type indicates statistical significance.

**Table 5:** Multivariate Cox regression analyses with regard to 30-day all-cause mortality within the entire study cohort.

Variables	HR	95% CI	*p* Value
**Model 1**			
Age (years)	1.016	1.001–1.031	** 0.038 **
Male sex	1.222	0.824–1.810	0.319
Heart rate (bpm)	1.006	0.999–1.013	0.083
Norepinephrine (µg/kg/min)	1.111	0.911–1.356	0.297
Lactate (mmol/L)	1.084	1.036–1.134	** 0.001 **
Creatinine (mg/dL)	1.138	1.013–1.279	** 0.030 **
Cardiopulmonary resuscitation	1.595	1.211–2.101	** 0.001 **
cTNI > 0.763 µg/L	1.915	1.298–2.824	** 0.001 **
Model 2 *			
cTNI > 0.763 µg/L	2.822	1.487–5.356	** 0.002 **
NT-proBNP > 4089 pg/mL	1.295	0.669–2.507	0.443

cTNI, cardiac troponin I; NT-proBNP, aminoterminal pro-B-type natriuretic peptide. Level of significance *p* < 0.05; bold type indicates statistical significance. * Model 2: lultivariable models were re-calculated after additional multivariable adjustment for NT-proBNP levels.

**Table 6:** Multivariate Cox regression analyses with regard to 30-day all-cause mortality stratified by non-AMI and AMI-related CS.

Variables	Non-AMI-Related CS			AMI-Related CS		
	HR	95% CI	*p* Value	HR	95% CI	*p* Value
Age (years)	1.014	0.989–1.041	0.278	1.011	0.989–1.033	0.327
Male sex	1.293	0.708–2.364	0.403	1.228	0.672–2.244	0.505
Heart rate (bpm)	1.001	0.991–1.011	0.889	1.010	0.999–1.022	0.065
Norepinephrine (µg/kg/min)	0.843	0.566–1.255	0.400	1.248	1.009–1.543	** 0.041 **
Lactate (mmol/L)	1.123	1.047–1.204	** 0.001 **	1.080	1.019–1.144	** 0.009 **
Creatinine (mg/dL)	1.187	0.991–1.421	0.063	1.101	0.933–1.299	0.257
Cardiopulmonary resuscitation	2.006	1.290–3.118	** 0.002 **	1.423	0.976–2.077	0.067
cTNI > 0.763 µg/L	1.953	1.021–3.735	** 0.043 **	1.807	1.019–3.202	** 0.043 **

cTNI, cardiac troponin I. Level of significance *p* < 0.05; bold type indicates statistical significance.

## Data Availability

The datasets used and/or analyzed during the current study are available from the corresponding author upon reasonable request.
